# Stress and behavior patterns throughout medical education – a six year longitudinal study

**DOI:** 10.1186/s12909-021-02862-x

**Published:** 2021-08-28

**Authors:** Edgar Voltmer, Susen Köslich-Strumann, Jan-Bennet Voltmer, Thomas Kötter

**Affiliations:** 1 Institute of Social Medicine and Epidemiology, University of Lübeck, Ratzeburger Allee 160, Lübeck, 23562 Germany; 2 Department of Psychology/Social Psychology, Distant-Learning University (FernUniversität) Hagen, Universitätsstraße 47, Hagen, 58097 Germany; 3 Institute of Family Medicine, University Medical Centre Schleswig-Holstein, Ratzeburger Allee 160, Lübeck, 23562 Germany

**Keywords:** Students, Medical, Health behavior, Burnout, Coping, Health promotion

## Abstract

**Background:**

Medical education has a reputation for being demanding and stressful. However, longitudinal surveys across the whole course of study considering risks and resources are rare.

**Methods:**

For the evaluation of stress and coping we administered the standard instruments Perceived Medical School Stress Scale (PMSS), Hospital Anxiety and Depression Scale (HADS), Work-Related Behavior and Experience Patterns (AVEM), Maslach Burnout Inventory (MBI), and a short form of the Coping Orientations to Problems Experienced Scale (Brief COPE) in three consecutive cohorts of medical students (*N* = 377) at one German university. Students were surveyed at the beginning of their studies (t0) and again during each consecutive summer semester (t1-t6).

**Results:**

Stress and symptoms of anxiety and depression increased in the first two years of medical studies but decreased again towards their end. Consistently, freshmen medical students presented with a large proportion of the healthy pattern at t0 (56 %) that decreased to 30 % at t2, and increased up to 44 % at t6. Correspondingly, the proportion with the burnout-related risk pattern B increased from 9 to 16 % at t2, again decreasing to 7 % at t6. Over the whole course of study there was an almost continuous increase of the unambitious pattern S from t0 13 to 40 % at t6. Characteristic differences especially between the healthy pattern and the risk patterns regarding stress, mental health symptoms and coping were observed. Female students showed a higher vulnerability for stress, anxiety and depression as well as lower proportions with a healthy pattern, and higher proportions with risk patterns for overexertion and burnout.

**Conclusions:**

The development of stress, symptoms and behavior and experience patterns especially in the first two years, demonstrating increasing study-related stress in the preclinical years, as well as the high proportion with an unambitious pattern at the end of the course of study emphasize the need for prevention and health promotion at both the individual and contextual levels.

## Background

Medical education has a reputation for being one of the most stressful courses to study. High workload, strict absence rules, frequent examinations, and increasing responsibility for patients are repeatedly named by students as sources of stress [1, 2]. Numerous cross-sectional studies have proven high levels of stress, anxiety, depression, and burnout at different time points of medical education [3–6]. A study at seven US medical schools reported that 47 % of students were positively screened for depressive symptoms, 50 % for burnout, and 25 % agreed on having ever considered suicide [7]. There is evidence that depression and burnout may impair performance in the course of study and quality of patient care in later work-life [8, 9].

Surveys in US physicians compared to the general population revealed that a higher proportion of physicians were dissatisfied with their work life balance and a higher and increasing proportion presented with burnout symptoms [10]. This is not only a question of personal wellbeing. Approximately $4.6 billion in costs were estimated as a result of physician turnover and reduced clinical hours that could be attributed to burnout [11]. Various researchers accredit the roots of this later development to the course of medical education [12, 13]. Contextual factors of institutions (e.g., university) like the learning climate, the amount of content to be learned, or the frequent examinations as well as a competitive medical culture have to be taken into account; this is in addition to factors of individual behavior like setting the personal bar very high [14–17]. It has to be noted critically that medical culture is not supportive of those seeking professional help in times of personal stress [16, 18].

With the identification of emotional exhaustion, cynicism, and reduced personal accomplishment both the concept and the measurement of burnout (the measure most predominantly used is the Maslach Burnout Inventory (MBI) [19]) follow a pathogenic perspective that asks for factors causing illness. In contrast, Antonovsky [20] developed a salutogenic approach that questions what keeps people healthy. It describes resources of resistance and the sense of coherence (SOC), a personal conviction that one is able to handle the challenges of daily life and that things will go as well as could be reasonably expected. The SOC is supported by perceptions of comprehensibility, manageability, and meaning. Based on this concept, Schaarschmidt and Fischer [21] developed the instrument “Work-Related Behavior and Experience Pattern (Arbeitsbezogenes Verhaltens- und Erlebensmuster, AVEM)” that not only identifies a behavior and experience pattern at risk for burnout (risk pattern B) but also a healthy pattern (G), a pattern with low working motivation (pattern S), and a pattern at risk for overexertion (risk pattern A; for a further description see Method section). These patterns could be perceived as indicators and also as reactions of students to study-related strain and offer an opportunity for the development of tailored measures of health promotion. In a series of cross-sectional studies in medical students and physicians in their early professional years a decrease in the healthy pattern and a corresponding increase in the burnout-related pattern has been reported [22]. At the end of the study and in early work-life a large proportion of students and physicians presented with an unambitious pattern characterized by reduced working motivation. Closely related to the stressful studies and resulting impairments of health is the question of how to handle this psychosocial stress to prevent medical students and later physicians from unhealthy occupational experiences and behaviors.

Based not least on the transactional stress model of Lazarus and Folkman [23], Carver et al. [24] analyzed different coping strategies for dealing with stress [25]. The coping reactions were divided into those that seemed to be generally adaptive/functional (e.g., active coping/planning, positive reframing, humor) while others might be considered as problematic/dysfunctional (e.g., denial, substance use, self-blame). Frost and Mierke [26] in their examination of students with a risk pattern comparable to risk pattern A found lower scores of functional and higher scores of dysfunctional coping reactions.

For female students a higher vulnerability regarding study-related stress has been reported [27, 28]. In female medical students depression, anxiety, somatization, and prevalent psychotropic substance use were found to a higher degree than in their male counterparts [29]. There may also be different preferences to cope with stress between genders. Daughtry and Paulk [30] identified a broader range of coping strategies in women who also seemed to practice avoidance-focused techniques and emotional-oriented coping more often [27, 31]. These differences have to be further analyzed not least with regard to the fact that in Germany about two thirds of freshman medical students are female [32].

There are some longitudinal surveys, usually covering shorter periods of time of one [7, 33] or two years [34], indicating a progression of psychosocial stress during the course of study. Longitudinal studies over the whole course of medical education are rare, reporting decreasing life satisfaction and ambiguous effects of different coping strategies [35, 36]. In a recent longitudinal study in medical students over the first three years of study, a decrease in the proportion of students with the healthy pattern and an increase in those with a burnout pattern was reported [37]. The present study seeks (1) to extend the examination of medical students’ development of stress, mental health symptoms, and the four behavior and experience patterns (AVEM) over the complete course of study and (2) to expand the evidence of medical students’ psychosocial conditions by investigating associations of the four different behavior and experience patterns to mental health symptoms (stress, anxiety, and depression) and associated coping behavior.

## Method

### Study design and setting

Data was drawn from an ongoing prospective, longitudinal observational study at the University of Lübeck [28, 38]. Each year, about 185 medical students start their course of study at the University of Lübeck. From 2011 onwards, all freshmen were invited to participate. The baseline surveys (t0) were taken in class during the pre-course week (prior to the beginning of courses). The follow-up surveys were taken online in June during the respective summer semesters. A €5 book or food voucher per completed questionnaire was used as an incentive for participants. For this study, three cohorts (2011–2013) of medical students were followed through the whole course of study (t0-t6). For the identification of datasets in the longitudinal analyses the participants were asked to generate a personal identification code and/or provide their matriculation number.

### Instruments

At each measurement point the questionnaires included multiple items related to different aspects of the study program. In the following section, only the measures relevant to the present analyses will be described. If not stated otherwise, items were recoded so that higher scores indicated higher values on the respective constructs.

#### Perceived Medical School Stress Scale (PMSS-D; t1-t6)

The Perceived Medical School Stress Scale comprises 13 items [16] rated on a five-point Likert scale (1 = I strongly disagree; 5 = I strongly agree). The items address participants’ perceptions of medical school (e.g., “The study of medicine promotes feelings of anonymity and isolation among students.”) and worries about work and competencies (e.g., “I worry that I will not be able to stand the long working hours and the responsibility that comes with clinical training and practice.”) as well as finance and accommodation (e.g., “My financial situation worries me.”). For each participant we calculated their total PMSS-D score by averaging the 13 items (0.79 ≤ Cronbach’s α ≤ 0.86).

#### Hospital Anxiety and Depression Scale (HADS-D; t0-t6)

The instrument was primarily developed for the detection of symptoms of anxiety and depression in clinical settings but has proved to be useful and appropriate in non-clinical settings too [39–41]. The instrument that we used in the German version comprises 7 items on anxiety (e.g., “I feel tense and strained”) and 7 items on depression (e.g., “I feel slowed down in my activities”), all rated on a four-point Likert scale answering format with higher values indicating higher anxiety/depression. We calculated total average and sum scores for the respective items (anxiety 0.74 ≤ Cronbach’s α ≤ 0.86; depression 0.77 ≤ α ≤ 0.83). Cut off sum scores were defined as < 7 per subscale = “safely without affection”, 8 to 10 = “borderline”, 11 to 14 = “severe”, and 15 to 21 = “very severe symptomatic” [39].

#### Work-Related Behavior and Experience Pattern (AVEM; t0-t6)

We report here the results of the 44-item short form of the standard instrument: “Work-Related Behavior and Experience Pattern [Arbeitsbezogenes Verhaltens- und Erlebensmuster, AVEM] [21]. On the basis of eleven health relevant dimensions from the domains of professional ambition, resistance toward stress, and emotional wellbeing (in the context of work/study) this instrument allows researchers to identify four health relevant behavior and experience patterns:

##### Pattern G (healthy)

Students with this pattern are characterized by a good balance between study-related ambition, resistance toward stress, and emotional wellbeing.

##### Pattern S (unambitious)

Compared to the healthy pattern G, a significantly lower study-related ambition characterizes this pattern, whereas resistance toward stress and emotional wellbeing remain positive.

##### Risk pattern A (overexertion)

Very high scores in the dimensions of study-related ambition but a poor ability to distance oneself from work/study and impaired emotional wellbeing characterize students with this pattern.

##### Risk pattern B (burnout)

With reduced ambition, poor resistance towards stress, and impaired emotional wellbeing this pattern represents the core symptoms of burnout syndrome [37, 42].

#### Maslach Burnout Inventory (MBI-SS; t5)

In this study we used the student-adapted version containing 15 items rated on a seven-point Likert scale [43, 44]. It comprises the subscales emotional exhaustion (5 items, e.g., “I feel exhausted from my studies.”), cynicism (4 items, e.g., “I have become more cynical about the potential usefulness of my studies.”), and efficacy (6 items, e.g., “I can effectively solve the problems that arise in my studies.”). For each subscale, we calculated the mean of the respective items for every participant (exhaustion, Cronbach’s α = 0.88, cynicism, α = 0.87, efficacy, α = 0.85).

#### Brief COPE Inventory (t5)

We used the short version of the “Coping Orientations to Problems Experienced Scale (COPE) [25]” with 14 scales and 28 items rated on a four point Likert Scale (Brief COPE) [45]. The items of each subscale correlated with 0.17 *≤* r ≤ 85. Following Meyer [46] we combined acceptance, emotional support, humor, positive reframing, active coping, instrumental support, planning, and religion as functional and behavioral disengagement, denial, distraction, self-blame, substance use, and venting as dysfunctional coping.

For demographics, age, gender, family status, and cohort membership were included.

### Data analysis

Data analyses were conducted with R and SPSS for Windows, Version 22.0 (IBM Corp., Armonk, NY, USA). Results of categorical analyses were reported as percentages. Preliminarily, we compared the three cohorts regarding sociodemographic characteristics using χ² tests. Cohort differences in AVEM patterns were analyzed using cohort membership as a predictor in hierarchical mixed modeling. In our main analyses we first used hierarchical linear mixed modeling [47] to investigate the development of stress, anxiety, and depression over time, and logistic hierarchical linear mixed modeling to investigate the occurrence of the four behavior and experience patterns over time. In the subsequent steps of the analyses, we added gender and sample/panel membership as predictors to investigate gender differences and differences between those who participated at all timepoints and those who participated only occasionally. Second, we added participants’ AVEM patterns to the models as predictors for stress, anxiety, and depression. The differences between the patterns were further analyzed using Tukey-adjusted post-hoc tests [48]. We conducted student’s t-tests investigating gender differences for burnout and coping, as well as ordinary least squares linear modeling with burnout and coping mechanisms as potential outcomes of AVEM patterns, further investigating differences between the patterns again using Tukey-adjusted post-hoc tests.

## Results

Of the three cohorts, a total sample of 377 participants at t0 was included in the longitudinal analysis (approximately 68 % of the presumed 185 freshmen per semester). The three cohorts were combined, since they did not differ regarding the relevant sociodemographics gender, age, or family status, and response rates over time (χ²(12) = 3.26, *p* = .99), nor did cohort membership add to the prediction of AVEM patterns at t0 (χ²(6) = 0.42, *p* = .99). Only students who had participated in t0 were included in the sample (t3 *n* = 237, t6 *n* = 141). Eighty-four students had participated in all 7 surveys (further referred to as “panel” in contrast to “sample”). When burnout and coping strategies were assessed at t5, 200 of those who had participated at t0 were still present in the data. Overall, 30 % of medical students who participated were male, on average 21.0 (*SD* = 3.1) years old, and predominantly either single (63 %) or in a relationship (34 %) at t0.

### Stress, anxiety, and depression

There was a slight increase in perceived stress from t1 (*M* = 2.28, *SD* = 0.46) to t2 (*M* = 2.49, *SD* = 0.58) with a second smaller peak at t4 (Table 1). The same holds true for the development of the anxiety and depression scores. In mixed linear models with random intercepts and timepoints nested in participants (comparing linear, quadratic, cubic, and quartic trends) we explored the (non-linear) trend of stress, anxiety, and depression over time. The prediction of stress, anxiety, and depression was improved by adding quadratic trends (stress: *b* = -0.12, *t*(1021) = -3.87, *p* < .001; anxiety: *b* = -0.09, *t*(1351) = -6.10, *p* < .001; depression: *b* = -0.06, *t*(1351) = -4.92, *p* < .001) and cubic trends (stress: *b* = 0.01, *t*(1021) = 3.10, *p* = .002; anxiety: *b* = 0.01, *t*(1351) = 4.71, *p* < .001; depression: *b* = 0.01, *t*(1351) =, *p* < .001) to the linear trends of time (stress: *b* = 0.45, *t*(1021) = 4.86, *p* < .001; anxiety: *b* = 0.26, *t*(1351) = 7.33, *p* < .001; depression: *b* = 0.16, *t*(1351) = 5.82, *p* < .001), all *p*s ≤ 0.002. This supports the hypothesized pre-clinical increase in stress and later relief (see Table 1).

**Table 1:** Average Stress (PMSS-D), Anxiety (HADS Anxiety), Depression (HADS Depression) per timepoint

	t0	t1	t2	t3	t4	t5	t6
	M (SD)	M (SD)	M (SD)	M (SD)	M (SD)	M (SD)	M (SD)
Stress	-	2.28 (0.46)	2.49 (0.58)***	2.36 (0.55)**	2.47 (0.62)**	2.34 (0.55)***	2.30 (0.60)
Anxiety	0.84 (0.48)	0.93 (0.52)*	1.17 (0.56)***	0.86 (0.56)***	0.90 (0.58)	0.83 (0.55)	0.75 (0.53)
Depression	0.42 (0.37)	0.47 (0.39)	0.60 (0.45)***	0.43 (0.42)***	0.44 (0.42)	0.36 (0.36)	0.34 (0.35)

Differences were examined using post-hoc comparisons in mixed linear models with time-point as predictor and stress, and anxiety or depression as outcome. Significant differences of the subsequent value compared to the preceding value are indicated at the subsequent value

*p < .05, **p < .01, ***p < .001

In clinical terms: Before medical studies, 72 % of the students could be considered “safely without affection [39]” regarding anxiety, and 95 % regarding depression. At t2, however, these values had decreased to 47 and 87 %, but recovered towards the end of the study period.

Adding gender as a predictor in mixed linear models significantly improved the prediction of stress (χ²(1) = 5.59, *p* = .02) and anxiety (χ²(1) = 9.50, *p* = .002). Male participants on average perceived less stress (b = -0.13, t(1020) = -2.36, *p* = .02) and anxiety (b = -0.15, t(1350) = -3.09, *p* = .002) during the course of their medical studies. In contrast, no differences existed regarding depression (χ²(1) = 0.56, *p* = .46).

### Development of behavior and experience patterns

**Fig. 1:**
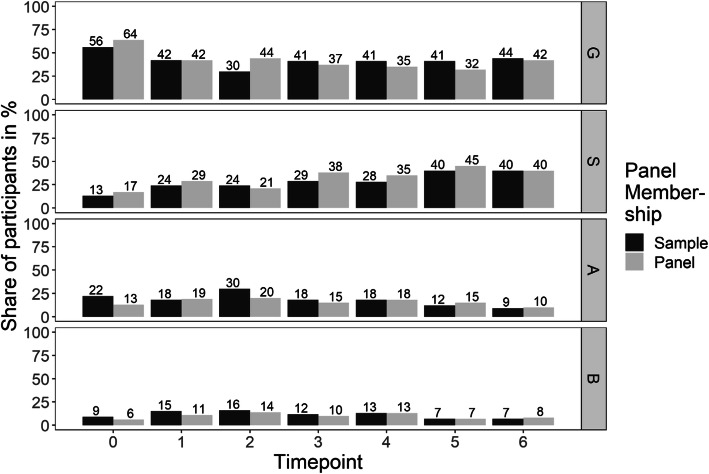
Health-related behavior and experience patterns over 6 years from all medical students who participated in t0 (sample) and from those students who participated at all time points (panel; *n* = 84). G = pattern G (healthy), S = pattern S (unambitious), A = risk pattern A (overexertion), B = risk pattern B (burnout)

A large proportion of freshmen medical students (sample) presented with the healthy pattern (56 %) that decreased to 30 % at t2, again increasing up to 44 % at t6 (see Fig. 1). Correspondingly, the proportion with the burnout-related risk pattern B increased from 9 to 16 % at t2, again decreasing to 7 % at t6. Over the whole course of study there was an almost continuous increase of the unambitious pattern S from t0 at 13 to 40 % at t6. Neither the sample nor the panel differed in the occurrence of the four patterns over time in four separate logistic regression models.

**Fig. 2:**
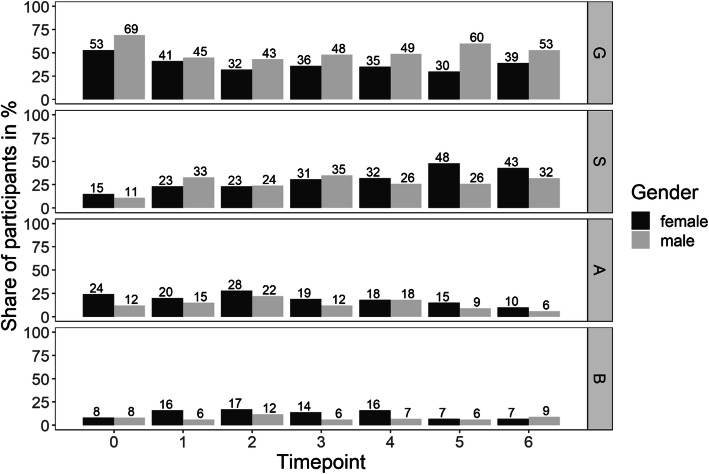
Health-related behavior and experience patterns over 6 years from all medical students who participated in t0 (sample) for female (f) and male (m) students. G = pattern G (healthy), S = pattern S (unambitious), A = risk pattern A (overexertion), B = risk pattern B (burnout)

As before adding gender as a predictor significantly improved the prediction of presenting with the healthy pattern (χ²(2) = 16.7, *p* < .001) with female participants presenting significantly less often with the healthy pattern than male participants averaged over all timepoints (*b* = -0.70, *se* = 0.31, *z* = -2.19, *p* = .03; Fig. 2). Instead at most timepoints female students presented with higher proportions of the risk patterns.

Taken together for the first two pre-clinical years of medical education we found a significant increase in stress and mental health symptoms as well as the proportion of students with risk patterns (AVEM). In the following clinical years this development partly reversed but a high proportion of students with the unambitious pattern S emerged.

### Differences among the four behavior and experience patterns in stress, anxiety, depression, burnout, and coping parameters

**Fig. 3:**
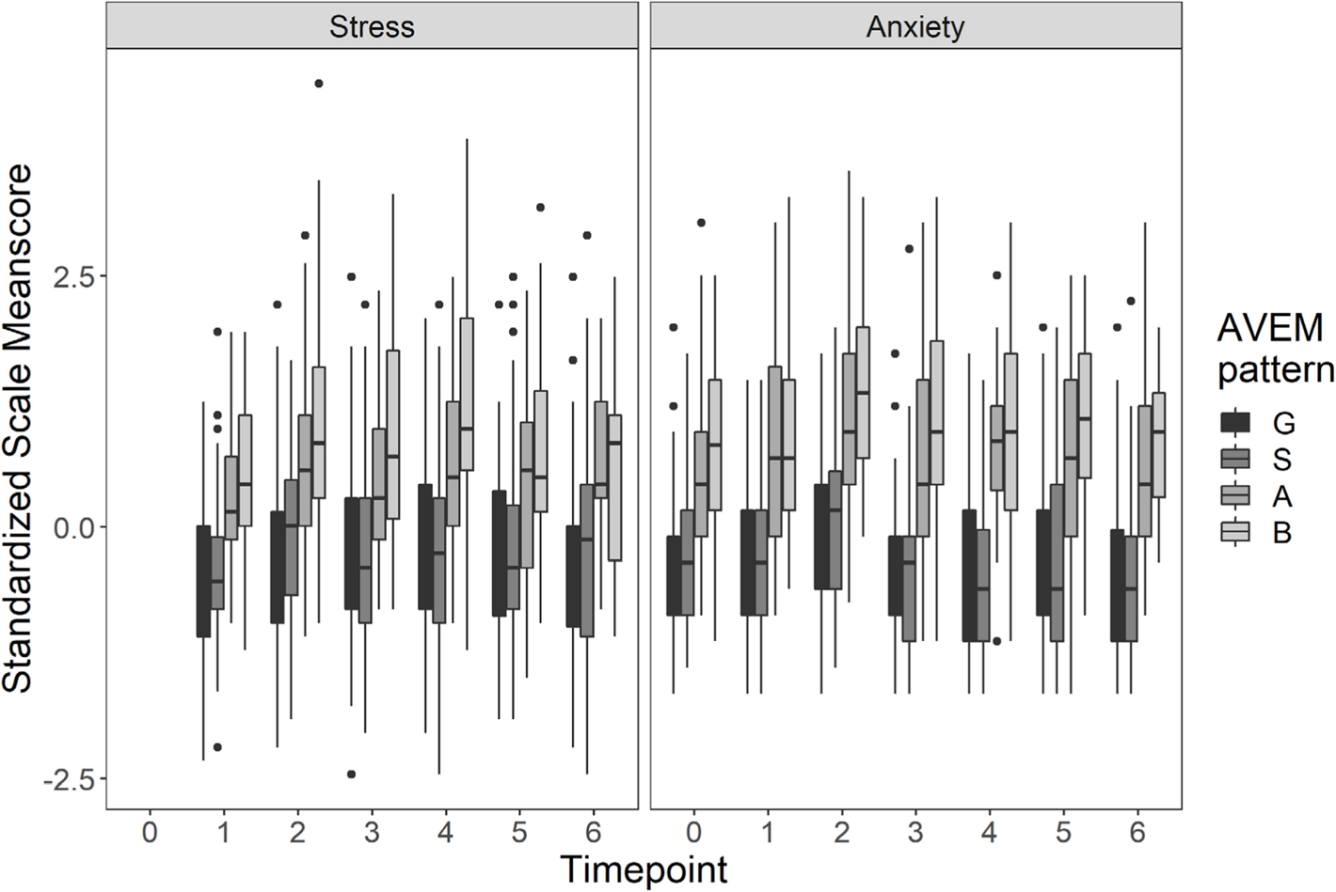
Differences in AVEM patterns in perceived stress and anxiety during the course of study (all medical students who participated in t0 (sample); G = pattern G (healthy), S = pattern S (unambitious), A = risk pattern A (overexertion), B = risk pattern B (burnout)

Throughout the whole course of study students with the risk pattern for burnout presented with significantly higher scores in stress, anxiety, and depression than those participants in the other patterns (Fig. 3). Adding the AVEM as an additional predictor to the aforementioned mixed linear models revealed significant differences in the AVEM patterns regarding stress, anxiety, and depression (χ²s(1) ≥ 128.61, ps ≤ 0.001). Since no interactions between timepoint and AVEM pattern took place, that is, the stress, anxiety, and depression differences did not differ between timepoints, we analyzed the average differences between the AVEM patterns over time in the next step. These post-hoc analyses revealed that significant differences existed between all patterns (stress: bs ≤ -0.17, t(1014) ≤ -4.01, ps ≤ 0.001; anxiety: bs ≤ -0.11, t(1344) ≤ -2.92, ps ≤ 0.02; depression: bs ≤ -0.14, t(1344) ≤ -4.54, ps ≤ 0.001) with one exception: The healthy and the unambitious patterns did not differ either regarding stress, anxiety, or depression.

MBI and Brief COPE were administered at t5. Students with the healthy pattern G scored highest to a significant degree in the efficacy scale and lowest in the cynicism scale (Table 2). Students with the risk pattern for burnout scored lowest in efficacy and significantly higher than students with the healthy pattern G and the unambitious pattern S in exhaustion. They also achieved the highest scores in the cynicism scale. Students with the healthy pattern G and the unambitious pattern S did not differ significantly in terms of exhaustion. This also holds true for those with the risk patterns A for overexertion and B for burnout. However, the students with the risk patterns had significantly higher exhaustion scores than those with patterns G and S. Consistently, students with the risk pattern for burnout scored lowest in functional but highest in dysfunctional coping (Table 2).

**Table 2:** Differences of behavior and experience patterns in MBI and Brief COPE subscales at t5

	Pattern G: Health	Pattern S: Unambitious	Risk pattern A: Overexertion	Risk pattern B: Burnout	*P* < .05
	M (SD)	M (SD)	M (SD)	M (SD)	
Exhaustion	3.09 (0.89)	3.05 (0.95)	4.32 (1.13)	4.31 (1.42)	G-A, G-B, S-A, S-B
Cynicism	1.85 (1.00)	2.26 (1.33)	2.44 (1.22)	3.45 (2.03)	G-S, G-A, G-B, S-B, A-B
Efficacy	5.57 (0.79)	4.80 (1.01)	4.93 (0.91)	4.01 (0.73)	G-S, G-A, G-B, S-B, A-B
Functional coping	2.87 (0.35)	2.81 (0.35)	2.50 (0.45)	2.43 (0.39)	G-A, G-B, S-A, S-B
Dysfunctional coping	1.86 (0.26)	1.90 (0.25)	2.01 (0.26)	2.15 (0.40)	G-B, S-B

## Discussion

In our prospective, longitudinal study following medical students from the start of their courses up to the end we analyzed the development of stress, mental health symptoms, and four behavior and experience patterns (AVEM). To expand the understanding of the pattern characteristics the associations of the patterns to study-related stress, anxiety, and depression as well as coping strategies are reported.

The distribution of patterns as well as the scores of stress, anxiety, and depression indicate an increasing perception of stress especially over the first two pre-clinical years of medical studies followed by a later decrease in the four clinical years, especially considering the risk patterns for overexertion and burnout. Other studies affirm the perception of increasing psychosocial strain in the course of medical education [22, 34, 35] and confirm stress to be a significant predictor of anxiety and depression [49]. The development in the first two pre-clinical years seems to be mainly determined by the major examination after the second year in the German structure (first part of the medical examinations, M1). In the clinical years there is a second, albeit lower, peak at t4 followed by a later decrease up to the sixth year; this is particularly true for the risk patterns for overexertion and burnout. The second peak might be influenced by the fact that with pharmacology and microbiology there are still a further two theoretical clinical subjects with a high amount of material to be learned. The perceived stress and the negative consequences for mental health in the early years of medical studies have also been reported by studies outside of Germany [29, 50, 51]. This is also the case for the slight relaxation in clinical years; stress in second year Viennese medical students was higher than in sixth year students [14]. Possible reasons may be the greater practical relevance of the topics as well as a greater experience in dealing with the amount of content to be learned and examination stress. However, the high proportion of students with an unambitious pattern at the end of the course of study must be noted critically. At the University of Lübeck a traditional course concept is applied with natural sciences and anatomy concentrated in the first two years with less patient contact in theory and practice than in reformed study organization. In reformed models the examination is often divided into more than one part and distributed over (later) timepoints [52, 53]. While these reforms are evaluated regarding performance, the psychosocial strain is less frequently addressed. One of the rare insights from a comparison of first year medicals students indicates a perception of less strain and competition in reform students [54]. In a 2014 report the Science Council of the German Government recommended the early integration of patient contact and a stronger bond between basic sciences and clinical specialties. It also advocated a revision of examination practices [53].

The analysis of the pattern differences in stress, anxiety, and depression revealed that in particular students with risk patterns for burnout but also overexertion are more affected than those with a healthy or unambitious pattern. This was also shown in a study of students in different courses of education from different universities [26]. Students with a risk pattern had lower scores of self-efficacy and internal locus of control. These differences between patterns have also been observed in professional spheres. In teachers those with a risk pattern for burnout or overexertion had the highest level of mental health symptoms, including anxiety and depression [55]. Entrepreneurs evidencing the risk pattern of overexertion scored significantly lower in self-confidence and personal health care than those with the healthy pattern [56].

Additionally MBI scores were more critical in students with a risk pattern. The emotional exhaustion scale of the MBI is most validated for the clinical relevance of the burnout syndrome. In our study there were significant differences in emotional exhaustion between but not within students with risk patterns (A and B) and those with non-risk patterns (G and S). This could reasonably be expected by the described characteristics of the patterns and underlines the general distinction between risk- and non-risk patterns. It was also one reason why, in the initial description of the AVEM instrument, the unambitious pattern S was not seen as a risk constellation for health but more as an issue of working motivation. However results of longitudinal studies demonstrate that a substantial amount of the participants with the unambitious pattern S changed to the risk pattern B [34]. The differences within the risk- and non-risk groups in the other two subscales of the MBI are therefore noteworthy (see below). Given the widespread distribution of the MBI it is important to notice that the high scores of emotional exhaustion in students with the AVEM risk pattern for burnout demonstrate a good accordance between both instruments. However with the four AVEM patterns there is additional value because on the one hand the scores of students with risk pattern A were comparably high and demonstrate the costs of constant overexertion. Consistently, in the AVEM systematic, to burn out would be characterized by the transition from A to B. On the other hand, on the cynicism scale, students with the risk pattern for burnout also presented with the highest scores of all patterns and even significantly higher than students with the risk pattern A. This emphasizes the discriminant validity of the risk patterns for certain phenomena. It has to be noted though that the student version of the MBI mainly addressed cynical perceptions regarding the course of study while the MBI for the general population focusses on interpersonal/patient contact. With this in mind it underlines the critical perception of study-related stress. This also holds true for the third subscale of efficacy in which students with risk pattern B presented with the lowest scores again with a significant difference also to those with risk pattern A. These results about study-related stress and risk of burnout are supported by quite a number of studies in medical students using the MBI. Dependent on the cohort and study phase at risk student proportions of 10 % to almost 50 % were reported [7, 57, 58].

The differences in AVEM patterns were also seen in coping strategies. Students with the healthy pattern were more inclined to use functional coping strategies whereas those with the risk pattern for burnout were more inclined to use dysfunctional measures. This was also found in a group of students where those with the risk pattern scored higher in dysfunctional and lower in functional coping strategies [26]. Nielsen and Knardahl [59] emphasized the importance of patterns for health against the single dimension from their cluster analysis of coping patterns; they reported that participants in the disengagement coping cluster, which included self-blame and self-distraction, reported significantly higher levels of psychological distress at both baseline and follow-up. Despite this there was no main effect of coping on the level of distress. Aside from the AVEM typology it has been reported that medical students used more active than avoidant strategies [60] and that the higher use of active coping strategies decreased stress levels [14]. Our data support that the functional coping strategies of active coping as well as acceptance, emotional, and instrumental support by other people moderate the effects of stress on the development of burnout. Other studies prove that factors such as social support may buffer the effects of stress and job demands on the development of burnout in medical students [15] or physicians [61, 62].

The demonstrated differences between the behavior and experience patterns are vital to notice because the development of stress in student groups is usually described by mean scores of the total cohort. However, our analysis of the four AVEM patterns reveals that different student groups perceive stress and cope with it differently. In terms of health promotion this discredits a one-size-fits-all model. It seems to be important to assess the individual setup before administering measures of prevention and to tailor pattern-specific empowerment for student groups.

### Gender differences in the experience of stress and coping resources

A higher vulnerability to study-related stress and the development of psychosocial symptoms in female students has to date been proven by a multitude of studies and reviews [4, 14, 26, 60]. In a representative survey of German students 54 % of female students felt exhausted by study-related stress compared to only 35 % of their male counterparts. Symptoms that could be perceived as psychosomatic reactions to study-related stress like headache, backache, sleep disturbance, stomach ache, concentration disturbance, and frequent cold were consistently more often found in female than in male students [63]. In our results throughout the whole course of study female students were seen with lower proportions of the healthy pattern compared to their male counterparts. At most of the time points they had higher depression and anxiety scores and presented with the risk patterns for overexertion and burnout more often. Since in Germany more than two thirds of medical freshmen are female universities have to adjust programs of teaching, mentoring, and health promotion to the specific characteristics and needs of the learning and perception of this important target group [64]. A stigmatizing of gender-specific behavior patterns or a gender-related norming has to be prevented.

To take care of male and female study-related stress seems to be even more important since a number of studies agree that stress, anxiety, depression, and dysfunctional coping are often correlated with poor performance. For example high stress perceptions in examinations verified by saliva cortisol and amylase levels were negatively correlated with performance in nursing students [65]. In contrast medical students with lower stress levels presented with better grades in the first examination [66].

### Strengths and limitations

A strength of our research is that it is one of the rare longitudinal studies that follow medical students over the whole course of their study. We analyzed both longitudinal and cross-sectional data, and there was good alignment between them (see Fig. 1). The response rate of these medical students was good. Limitations include that the study is based on self-report instruments and a higher percentage of female medical students participated in the study than the estimated percentage of female medical students in total. The study was conducted in one German university and the generalizability of our results is thus limited. However the age and gender distribution among students at Lübeck Medical School resemble the nationwide distribution and the study place allocation is partly centralized. The local findings may therefore be generalized at least to a national level.

### Implications for health promotion and future research

In a remarkable editorial Roberts stated that “many of the experiences of medical school may overwhelm and exhaust rather than inspire and instruct students. Indeed, contrary to the intent of medical educators, the experiences of medical training may damage the well-being and diminish the professionalism of many early-career colleagues” [12]. The development of stress especially in the first two years of medical education in traditionally organized study raises questions about structural changes aiming at setting or institution specific factors such as those in reform concepts [52]. A stronger integration of practice and patient contact as well as practical and theoretical content might improve motivation and facilitate learning. However comparisons regarding psychosocial stress between reform and traditional concepts are still lacking. This also holds true for structural programs to reduce the stress of frequent examinations and to improve autonomy supportive teaching. Performance pressure was one of the two most important stressors in Viennese medical students [14]. A pass/fail system instead of a differentiated grading system significantly increased personal well-being and satisfaction with life [67]. Support from faculty members and a positive learning climate have also been effective in preventing burnout [15, 68] and may foster academic performance [54].

More important thus are efforts to change the culture at medical schools, e.g., by the foundation of health promotion advisory boards including teachers, students, and administration staff, aiming to create a healthy learning environment and reduce unnecessary medical school-associated stress [69]. Interventions at a contextual level may have a higher impact on medical students’ health and wellbeing than those (solely) aiming at the individual behavior [68].

Addressing the individual behavior, setting personal limits very high is another main stressor for students [14]. Small group Mind Body Medicine training seems to be a promising approach in addressing the needs of medical students and fostering sustainable coping strategies [70]. Meditation- and mindfulness-based interventions have also been shown to reduce stress and improve mood [71, 72]. An elective course for the learning of relaxation techniques (autogenous training, progressive muscle relaxation (PMR)) was effective in significantly reducing cognitive and emotional burnout as well as trait anxiety [73]. In first year medical students escape avoidance was related to lower levels of psychological well-being while positive reappraisal and planful problem-solving were related to higher levels of psychological well-being [74]. In a longitudinal US survey more than half of medical students did not seek the necessary treatment for health problems and an increasing proportion raised concerns about confidentiality (69 % in pre-clinical versus 83 % in clinical years) and fear of academic reprisal [75, 76]. Fostering a culture of transparent and blame free communication about personal health issues and offering low level support onsite or – even more importantly – in offsite settings may support medical students’ health and wellbeing.

The differences in student groups with different study-related behavior and experience patterns regarding stress, mental health symptoms, and coping prove a need to first select specific student groups and then address the specific needs in health promotion programs appropriately. This also holds true for the differences between male and female students. So far there is only limited evidence regarding gender-oriented interventions. Health promotion and prevention courses of health insurance companies in Germany are largely frequented by female participants (81 %) [77]. Beside anxiety being female was one of the strongest predictors for participating in a relaxation course of PMR in medical students [78]. Furthermore a PMR program reduced anxiety and improved quality of life in female medical students [79].

## Conclusions

The study set out to examine the development of psychosocial stress, mental health symptoms, and four behavior and experience patterns in medical students at one German university. The substantial and increasing fractions of students at risk for burnout, the decreasing fractions with a healthy study-related behavior and experience pattern especially in the preclinical years, and the increasing proportion of students with an unambitious pattern may emphasize the need for prevention and health promotion at both the individual and a contextual levels. Fostering this is likely to improve students’ health and performance.

## Data Availability

The datasets used and/or analysed during the current study are available from the corresponding author on reasonable request.

## Abbreviations

AVEM: “Work-Related Behavior and Experience Pattern” [Arbeitsbezogenes Verhaltens- und Erlebensmuster]
COPE: Coping Orientations to Problems Experienced Scale
HADS: Hospital Anxiety and Depression Scale
MBI: Maslach Burnout Inventory
